# Performance of disc diffusion and four commercially available MIC tests to determine mecillinam susceptibility on carbapenemase-producing Enterobacterales

**DOI:** 10.1128/jcm.01473-24

**Published:** 2025-04-14

**Authors:** Sacha Milleville, Lamia Rouabah, Sandrine Bernabeu, Anne Santerre Henriksen, Hippolyte De Swardt, Inès Rezzoug, Laurent Dortet, Cécile Emeraud

**Affiliations:** 1 Team Resist UMR1184 Immunology of Viral, Auto-Immune, Hematological and Bacterial diseases (IMVA-HB), INSERM, Université Paris-Saclay, CEA, LabEx LERMIT, Faculty of Medicine 89691, Le Kremlin-Bicêtre, Île-de-France, France; 2 Bacteriology-Hygiene Unit, Bicêtre Hospital, Assistance Publique-Hôpitaux de Paris 537860 , Le Kremlin-Bicêtre, Île-de-France, France; 3 Associated French National Reference Center for Antibiotic Resistance: Carbapenemase-Producing Enterobacteriaceae, Le Kremlin-Bicêtre, France; 4 Maxel Consulting ApS, Jyllinge, Denmark; Johns Hopkins University, Baltimore, Maryland, USA

**Keywords:** carbapenemase, mecillinam, testing method

## Abstract

Urinary tract infections (UTIs) are predominantly caused by Enterobacterales, and escalating resistance poses significant challenges due to carbapenemase production, which can compromise the efficacy of last-resort antibiotics. Pivmecillinam, as the prodrug of mecillinam, may serve as a treatment option for UTIs caused by carbapenemase-producing Enterobacterales (CPE). However, accurate methods are required to accurately assess susceptibility to this antibiotic. This study aimed to evaluate the *in vitro* susceptibility of mecillinam on a collection of 161 well-characterized CPE isolates collected from July to November 2023 in France and assess the performances of disc diffusion, Liofilchem MTS gradient strip, bioMérieux E-test, Sensititre broth microdilution, and VITEK 2 compared to agar dilution (reference method) in this specific population of CPE. None of the commercially available tests met the International Organization for Standardization standards with this entire collection. Using Clinical and Laboratory Standards Institute (CLSI) or European Committee on Antimicrobial Susceptibility Testing (EUCAST) guidelines on the complete collection of CPE, disc diffusion possessed the best categorical agreement [77.0 (124/161) or 84.5% (131/161), respectively]. Sensititre broth microdilution and VITEK 2 overestimated MICs (by 1.8 and one doubling-dilution, respectively) but had low very major error (VME) rates (0% (0/53) for Sensititre and 4.2% (6/53) for VITEK 2 with CLSI and 0% (0/53) for Sensititre and 11.3% (6/53) for VITEK 2 with EUCAST). Notably, when focusing solely on *E. coli* isolates (*n* = 45), performance improved considerably. Both disc diffusion and VITEK 2 demonstrated high categorical agreements [95.6 (43/45) and 93.3% (42/45), respectively] with no VME observed for either method using CLSI breakpoints. In conclusion, while agar dilution remains the reference method for mecillinam MIC determination, disc diffusion and VITEK2 are accurate alternatives to determine mecillinam susceptibility in CPE isolates, especially for *Escherichia coli*.

**IMPORTANCE:**

The rising prevalence of carbapenemase-producing Enterobacterales (CPE) poses significant challenges to effective antibiotic therapy, particularly for urinary tract infections. Pivmecillinam, the prodrug of mecillinam, offers a promising treatment option due to its activity against certain carbapenemase-producing strains. However, selecting accurate susceptibility testing methods is crucial for ensuring appropriate clinical use. This study rigorously evaluates the performances of various susceptibility testing methods against a challenging collection of CPE isolates, identifying the strengths and limitations of each. By providing critical insights into their reliability, particularly for routine clinical settings, this research supports improved diagnostic accuracy and optimal use of mecillinam in combating multidrug-resistant infections. These findings are especially relevant as pivmecillinam gains international approval, underscoring its potential as a valuable alternative in the fight against antibiotic resistance.

## INTRODUCTION

Urinary tract infections (UTIs) represent the most common infectious diseases encountered both in community and hospital settings (1). Among the causative agents, Enterobacterales predominate, especially *Escherichia coli* isolates. Unfortunately, antimicrobial resistance is an escalating issue, particularly regarding Enterobacterales that are resistant to carbapenems, which are considered as last-resort antibiotics. The carbapenem resistance typically arises either through the production of an extended-spectrum β-lactamase (ESBL) or a cephalosporinase coupled with loss of membrane permeability or through the production of carbapenemases. These carbapenemases can belong to three of the four Ambler classes: Amber class A (mainly KPC, IMI) (2), Amber class B (mainly NDM, VIM, IMP) (3), and Amber class D (OXA-48-like) (4). Amber class C primarily constitutes plasmidic or chromosomic cephalosporinases that exhibit limited or no activity toward carbapenems.

Pivmecillinam, recently approved by the US Food and Drug Administration for the treatment of adult women with uncomplicated UTIs, has been included as a first-line therapeutic option on the Infectious Diseases Society of America and European Society for Microbiology and Infectious Diseases guidelines since 2011 (5). This oral prodrug of mecillinam is rapidly hydrolyzed into its active form by unspecific esterases. Pharmacokinetic–pharmacodynamic studies have shown high liver, kidney, and prostatic diffusion, with an increased concentration in bile and urines reaching between 10 and 50 times minimal inhibitory concentrations (MICs) of susceptible microbiological agents (6, 7). The most recent European Committee on Antimicrobial Susceptibility Testing (EUCAST) guidelines (2024) have established breakpoints for mecillinam susceptibility testing in UTIs caused by *E. coli*, *Citrobacter* spp., *Klebsiella* spp., *Enterobacte*r spp., *P. mirabilis*, and *Raoultella* spp. These bacterial species often exhibit low MICs to this molecule. As an example, the epidemiological cut-off (ECOFF) of *E. coli* for mecillinam is 0.5 mg/L, while for fosfomycin it is 4 mg/L. However, few Enterobacterales species, such as *Morganella morganii* or *Serratia marcescens*, appear to be naturally resistant to mecillinam (8). In contrast, the Clinical and Laboratory Standards Institute (CLSI) guidelines are exclusively defined for *E. coli*.

Mecillinam has been used for more than 40 years in Nordic countries, but, despite this, resistance rates of *E. coli* to this antimicrobial remain below 6%, with defined daily doses per 1,000 inhabitants per day between 0.5 and 1.8 (9). Furthermore, mecillinam remains active against strains that produce cephalosporinase, ESBLs, or certain carbapenemases, such as OXA-48-like, NDM, or IMI. At the opposite end, VIM- or KPC-type carbapenemase producers are highly resistant (MIC > 256 mg/L) to mecillinam (8, 10–13).

The reference method for determining MICs to mecillinam is agar dilution. This method is cumbersome and not suitable for the high analytic volume of clinical microbiology laboratories. As pivmecillinam is a new therapeutic option in the USA that could also become a key antibiotic for treating infections caused by carbapenemase-producing Enterobacterales (CPE) in the community, it is crucial to assess all available methods for determining susceptibility and validate their accuracy. A previous study on multi-susceptible strains, particularly *E. coli*, showed that several methods for testing mecillinam susceptibility were effective (14). Here, we focused on carbapenemase-producing Enterobacterales from different species to evaluate whether these methods remain accurate for strains where mecillinam might be a treatment option. We tested the *in vitro* susceptibility of mecillinam on a collection of CPE using various clinical laboratory methods. Their effectiveness in categorizing strains as susceptible (MEC-S) or resistant (MEC-R) was assessed.

## MATERIALS AND METHODS

### Strain collection

This study included a total of 161 well-characterized CPE strains received at the French National Reference Center (F-NRC) from July 2023 to November 2023 mainly from rectal swab screening and urines. This collection included 45 *E. coli*, 43 *Citrobacter freundii*, 39 *Klebsiella pneumoniae*, 22 *Enterobacter cloacae* complex, and 12 other Enterobacterales isolates (Table S1). This collection included OXA-48-like (*n* = 88), NDM (*n* = 54), NDM + OXA-48-like (*n* = 10), VIM (*n* = 4), VIM + OXA-48-like (*n* = 3), KPC (*n* = 1), and KPC + NDM producers (*n* = 1). Strains of this collection were selected to fit a normal Gaussian distribution using the inhibition zones from the disc diffusion susceptibility testing initially performed at the F-NRC (Fig. S1A) in order to focus on strains that are challenging to categorize. Using the results previously obtained by disc diffusion, 70% (*n* = 124) of the isolates were considered susceptible to mecillinam.

### Mecillinam susceptibility testing methods

#### Disc diffusion

First, the inhibition diameters previously measured by the F-NRC were verified. For this purpose, a 10 µg disc of mecillinam (I2A, France) was placed on Mueller–Hinton agar (Bio-Rad, France) previously inoculated with a 0.5 McFarland bacterial suspension, and inhibition diameters were read after 16–24 h at 35°C. As recommended by the current EUCAST guidelines (not clearly specified in CLSI guidelines), colonies within the inhibition zone were not considered when measuring inhibition zone diameters.

#### MIC determination

MICs were determined using four different methods: agar dilution (the gold standard for EUCAST and CLSI), two different gradient strip tests, and one broth microdilution test.

For the agar dilution method (the gold standard), Mueller–Hinton agar (Beckton-Dickinson, France) was prepared with mecillinam concentrations ranging from 0.125 to 32 mg/L (Recipharm Brescia, Italy). Media were used fresh, and spots were inoculated with 10^4^ CFU/spot, and MICs were read after 16–24h of incubation at 37°C.

Mecillinam MICs were further determined by gradient strips (using Liofilchem test strip and bioMérieux E-test) and broth microdilution using Sensititre plates EUMDROXF (Thermo Fisher Scientific) according to the manufacturer’s recommendations.

#### Automated susceptibility testing

Finally, mecillinam susceptibility was determined using the VITEK 2 AST-N372 card (bioMérieux) according to the manufacturer’s recommendations.

Except for VITEK 2, which has its own recommendations for inoculum preparation, the same inoculum of 0.5 McFarland bacterial was used for all the other methods.

We used *E. coli* ATCC 25922 as the quality control strain, with MIC values for mecillinam by agar dilution (0.06 mg/L; expected range: 0.03–0.25 mg/L) and disk diffusion inhibition zones (29 mm; expected range: 24–30 mm) falling within the expected ranges.

The MICs or inhibition zone diameters were interpreted according to both EUCAST and CLSI recommendations as updated in 2024. According to EUCAST, the unique zone inhibition diameter breakpoint is 15 mm, corresponding to the MIC breakpoint of 8 mg/L. For *Serratia* and *Leclercia* isolates (*n* = 3/161), for which no EUCAST breakpoints are available, we applied the same breakpoints as for other Enterobacterales. According to CLSI, a zone inhibition diameter was considered susceptible if ≥15 mm, intermediate susceptibility between 12 and 14 mm, and resistant if inferior to 12 mm. For MICs, values of ≤8 mg/L were considered susceptible, 16 mg/L as indicative of intermediate susceptibility, and ≥32 mg/L as resistant. It is important to note that CLSI breakpoints are officially defined only for *E. coli*. Therefore, we also calculated the performance metrics both for all Enterobacterales using the CLSI breakpoints for *E. coli* and separately for *E. coli* isolates (*n* = 45) alone to align strictly with CLSI guidelines.

### Data analysis and statistics

For all methods, including the disc method, the rates of categorical agreement (CA), major errors (ME), and very major errors (VME) were calculated following the definition from ISO 20776-2:2007. Congruent expected performances were: CA and EA each at or above 90%, VME at or below 1.5%, and ME at or below 3% (15). As more than 20% of the isolates had MIC values within ±1 log2 dilution of the breakpoint(s), we also calculated the performance parameters using the error-rate-bound method (Table S2), as recommended by CLSI (16). Additionally, performance evaluations were conducted separately for the two main types of carbapenemases found in our data set: OXA-48-like and NDM (Table S2).

To compare MIC determination methods, as recommended in the International Organization for Standardization (ISO) 20776-2:2021 document, essential agreement (EA) and bias were calculated using the agar dilution method as gold standard. EA was defined as the percentage of isolates for which MIC was measured within ±one twofold dilution, as determined by the reference method. Congruent expected performances were: EA ≥ 90%, −30% ≤ bias ≤ +30%. Additionally, we conducted a non-parametric Wilcoxon rank-sum test on the log2-transformed data. The Wilcoxon statistic and *P*-values were calculated for each method, allowing us to assess significant differences between the methods and the reference.

Furthermore, we performed linear regression analysis on the log2-transformed data using Python (version 3.11) and the statsmodels library (version 0.13.1). For each method, we calculated the slope and intercept, along with their 95% confidence intervals. The slope indicates the correlation between the testing and reference methods, with a slope closer to 1, suggesting a stronger correlation. The *Y*-intercept indicates systematic bias, with values significantly different from 0, suggesting underestimation or overestimation by the method.

## RESULTS

First, when examining the distribution of MIC values determined using the reference agar dilution method, we confirmed a Gaussian distribution centered around the clinical cut-off value of 8 mg/L (Fig. S1B). According to the agar dilution reference method, 67.1% (*n* = 108/161) of the total CPE strains and 91.1% (*n* = 41/45) of the *E. coli* strains were categorized as MEC-S. Interestingly, when using CLSI recommendations, 18% of the total CPE strains (*n* = 29/161) were classified as intermediate (I), reducing the proportion of MEC-R strains from 32.9% (*n* = 53/161) according to EUCAST to 14.9% (*n* = 24/161) according to CLSI. As expected, the majority of OXA-48-like and NDM-producing strains were MEC-S (81% for OXA-48-like, 64% for NDM), whereas those producing VIM and KPC were MEC-R (100% for both) (8, 10–13).

Overall, regarding the other tested methods, 76.4 (*n* = 123/161), 96.9 (*n* = 156/161), 91.9 (*n* = 148/161), 23.6 (*n* = 38/161), and 47.8% (*n* = 77/161) of the strains were categorized MEC-S using the disc diffusion, Liofilchem gradient strip, bioMérieux E-test, Sensititre broth microdilution, and VITEK, respectively (Table 1).

**TABLE 1:** Comparative analysis of very major errors (VME), major errors (ME), minor errors (mE), bias, categorical agreement (CA), and essential agreement (EA) for broth microdilution, Liofilchem gradient strips, bioMérieux E-test, and VITEK 2 versus the reference agar dilution method^*a*^

		Agar dilution	Disc diffusion	Sensititre EUMDROFX broth microdilution	Liofilchem MTS strip	E-test bioMérieux	VITEK 2
		(Reference method)^*b*^					
EUCAST	% S (≥15 mm or ≤8 mg/L)	67.1%	76.4%	23.6%	96.9%	91.9%	47.8%
	% R (<15 mm or >8 mg/L)	32.9%	23.6%	76.4%	3.1%	8.1%	52.2%
CLSI all CPE	% S (≥15 mm or ≤8 mg/L)	67.1%	76.4%	23.6%	96.9%	91.9%	47.8%
	% I (12–14 mm or 16 mg/L)	18.0%	16.8%	24.8%	0.6%	1.9%	9.9%
	% R (<12 mm or >16 mg/L)	14.9%	6.8%	51.6%	2.5%	6.2%	42.3%
CLSI *E. coli*	% S (≥15 mm or ≤8 mg/L)	91.1%	91.1	46.7%	100%	95.6%	88.9%
	% I (12–14 mm or 16 mg/L)	4.4%	8.9%	20.0%	0%	4.4%	2.2%
	% R (<12 mm or >16 mg/L)	4.4%	0%	33.3%	0%	0%	8.9%
Comparison with the agar dilution reference method							
EUCAST	VME	-	37.7%	0%	90.6%	77.4%	11.3%
	ME	-	4.6%	64.8%	0.0%	0.9%	34.3%
	CA	-	84.5%	56.5%	70.2%	73.9%	73.3%
CLSI all CPE	VME	-	8.3%	0%	79.2%	54.2%	4.2%
	ME	-	0.9%	27.8%	0.0%	0.0%	25.9%
	mE		25.8%	52.3%	22.7%	24.2%	23.5%
	CA	-	77.0%	38.5%	69.6%	72.0%	62.7%
* *CLSI *E. coli*	VME	-	0%	0%	100%	50%	0%
	ME	-	0%	26.8%	0%	0%	0%
	mE		4.7%	25.6%	4.7%	4.7%	7%
	CA	-	95.6%	51.1%	91.1%	93.3%	93.3%
All CPE	EA	-	-	37.9%	3.7%	62.7%	62.1%
	Bias	-	-	+87.0%	−96.3%	−71.4%	+46.0%
*E. coli*	EA	-	-	22.2%	2.2%	77.8%	97.8%
	Bias	-	-	+95.6%	−97.8%	−60%	+42.2%
Slope (log2) and 95% CI		-	not tested	0.675	0.802	0.520	0.914
				[0.550; 0.801]	[0.661; 0.943]	[0.423; 0.617]	[0.892; 1.986]
Y-intercept (log2) and 95% CI		-	not tested	2.805	−3.402	−0.138	1.439
				[2.499; 3.112]	[−3.747 ;−3.057]	[−0.374 ;−0.099]	[0.892; 1.986]

^*a*^ S = susceptible, I = intermediate, R = resistant, and CPE = carbapenem-producing Enterobacterales.

^*b*^ "-" = Not applicable.

### Performances of disc diffusion to assess mecillinam susceptibility

**Fig 1:**
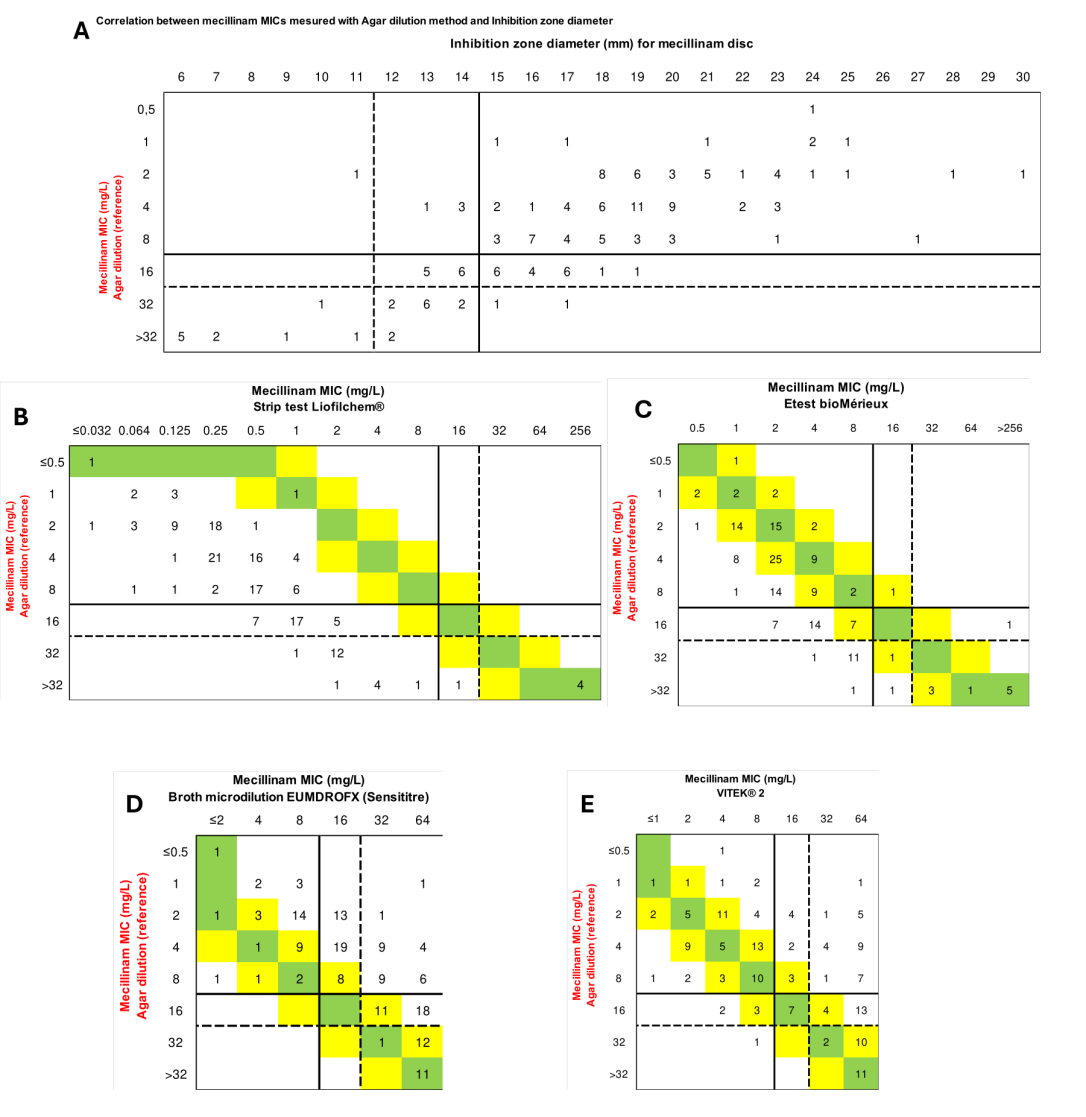
Comparison of different available methods determining mecillinam susceptibility with the reference method. (A) Correlation between agar dilution and disc diffusion methods. Comparison of MICs for mecillinam using Liofilchem MIC strip test (B), bioMérieux E-test (C), Sensititre broth microdilution (D), and VITEK 2 (E) compared to the reference method. Intermediate susceptibility zones (inhibition zone diameters between 12 and 14 mm and MIC at 8 mg/L) indicated according to CLSI recommendations are highlighted by a dotted line. Green cells indicate identical MIC values between the two methods, and yellow cells represent a ±1 doubling-dilution difference.

**Fig 2:**
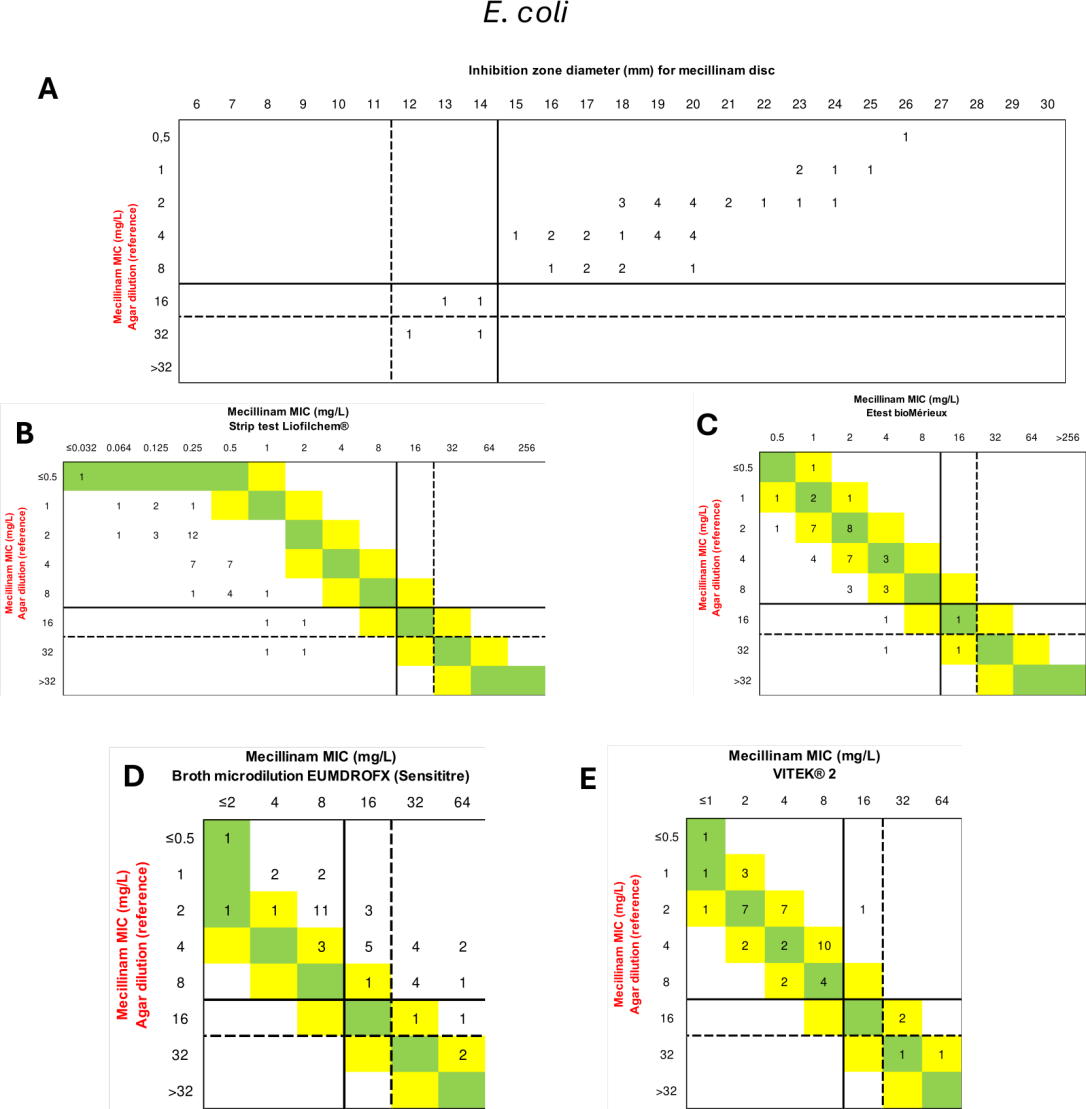
Comparison of different available methods determining mecillinam susceptibility with the reference method only for the 45 *E. coli* isolates included in this study. Correlation between agar dilution and disc diffusion methods (A). Comparison of MICs for mecillinam using Liofilchem MIC strip test (B), bioMérieux E-test (C), Sensititre broth microdilution (D), and VITEK 2 (E) compared to the reference method. Intermediate susceptibility zones (inhibition zone diameters between 12 and 14 mm and MIC at 8 mg/L) indicated according to CLSI recommendations are highlighted by a dotted line.

As shown in Table 1, despite disc diffusion having the higher CA (84.5% using EUCAST and 77.0% using CLSI breakpoints) of all tested methods, it did not reach the 90% requested for test validity on the total CPE collection (Table 1). Then, VME (=37.7%, *n* = 20/53) and ME (=4.6%, *n* = 5/161) did not reach the requested standards (VME ≤ 1.5% and ME ≤ 3%) using the EUCAST breakpoint (Fig. 1A; Table 1). Using CLSI guidelines, lower VME and ME were obtained, but the rate of VME remained too high (8.3%, *n* = 2/24) (Fig. 1A; Table 1). However, on the *E. coli* collection, VME and ME were both 0% (*n* = 0/2), and a high categorical agreement (CA) of 95.6% (*n* = 43/45) was obtained (Fig. 2A; Table 1). When applying the error-rate-bound method, CA reached ≥90% only for OXA-48-like-producing isolates according to EUCAST breakpoints (Table S2).

### Performances of Sensititre EUMDROXF AST broth microdilution plate, Liofilchem strip, biomérieux E-test, and VITEK 2 to determine mecillinam MICs

Regardless of the technique used, the EA remained under the requested standard (≥90%) with 37.3% (*n* = 60/161) for the Sensititre broth microdilution method, 3.7% (*n* = 6/161) for Liofilchem gradient strips, 47.2% (*n* = 75/161) for bioMérieux E-test, and 62.1% (*n* = 100/161) for VITEK 2 (Table 1).

Using a Wilcoxon rank test to compare the MICs obtained for the 161 CPE using agar dilution method (=reference), Liofilchem MTS strips, bioMérieux E-test, Sensititre EUMDROFX AST broth microdilution plate, and VITEK 2, we found significant differences in distributions. Indeed, compared to the reference method, the MICs determined with Liofilchem strip tests and bioMérieux E-test were significantly lower than those obtained with the reference method, while those obtained with Sensititre EUMDROFX AST broth microdilution plate and VITEK 2 were overestimated (*P*-value < 0.0001) (Fig. S2). These results were confirmed by the bias calculation that was below the requested −30% for both gradient strips (−96.3 and −71.4% for Liofilchem and bioMérieux, respectively) and over +30% for the Sensititre EUMDROFX AST broth microdilution plate (bias = +87.0%) (Table 1; Fig. 1B through E).

Statistically, the broth microdilution Sensititre method shows a slope of 0.675, indicating an overall low correlation with the agar dilution method, and a *Y*-intercept (log2) of 2.805, suggesting a slight overestimation at lower values. In contrast, the Liofilchem strip method exhibits a closer correlation with a slope of 0.802, but with a significant Y-intercept (log2) of −3.402, indicating substantial underestimation at the base level (Table 1). The bioMérieux E-test shows a slope of 0.520 with a Y-intercept (log2) of −0.138, indicating some underestimation at the base level. Finally, VITEK 2 shows a slope of 0.914 with a *Y*-intercept (log2) of 1.439, indicating a moderate alignment but with some overestimation (Table 1).

For all these methods, the CA ranged from 56.5 (*n* = 91/161) to 73.9% (*n* = 119/161) using the EUCAST breakpoint and from 38.5 (*n* = 62/161) to 72.0% (*n* = 116/161) using the CLSI breakpoints (Table 1; Fig. 1B through E). When applying the error-rate-bound method, regardless of the MIC determination method used, the reference system applied (EUCAST or CLSI), or the type of carbapenemase (all CPE, only OXA-48-like, or only NDM), none of the methods reached the expected performance criteria (Table S2).

However, when focusing on the *E. coli* collection, the CA exceeded 90% for all methods, except the broth microdilution method, which had a CA of 51.1% (*n* = 23/35) but with a VME at 100% (*n* = 2/2) for Liofilchem strips and at 50% (*n* = 1/2) for E-test strips. In contrast, VITEK showed excellent performance with both VME and ME at 0% (Fig. 2B through E, Table 1).

## DISCUSSION

Given the significant increase in the prevalence of antibiotic resistance, it is crucial to identify new therapeutic alternatives. Pivmecillinam represents a potential option, hence the need for accurate methods to determine mecillinam susceptibility in these resistant strains.

Our study focused on the performance of susceptibility testing methods for mecillinam using a challenging panel of carbapenemase-producing Enterobacterales isolates for validation. According to our comparative analysis, none of the methods tested fully met the performance criteria when using the full collection of CPE and the EUCAST or CLSI recommendations. However, the disc diffusion method interpreted according to CLSI guidelines comes closest to these criteria with an intermediate categorization at 12–14 mm and 16 mg/L that avoids many false classifications (Fig. 1A). Accordingly, among the methods evaluated, the disc diffusion technique commonly used in laboratories appears to be the least inadequate option.

Despite the overall overestimation of MICs using VITEK 2 and Sensititre EUMDROFX AST broth microdilution plate, these methods might still be used in laboratories, as they do not produce many false susceptible results (0 and 4.2% VME using CLSI guidelines for Sensititre EUMDROFX and VITEK 2, respectively). Accordingly, these two methods might be considered as efficient techniques to determine mecillinam susceptibility (but not resistance) in CPE isolates. However, the high rates of ME (>25%) led to the problem of categorizing a strain as resistant despite being susceptible, especially regarding the multidrug resistance phenotype of CPE leading to few therapeutic options.

As previously reported by Fuchs et al. (13), the most problematic methods remained as gradient strips (Liofilchem gradient strip and bioMérieux Etest), leading to the underestimation of the MIC and subsequent high rates of false susceptible categorizations (VME > 50% and > 75%

guidelines, respectively).

Nevertheless, the poor performance observed for disk diffusion, E-test, and VITEK 2 methods in this study is likely due to the high proportion of resistant isolates and the significant number of strains with MICs near the cut-off, increasing the risk of misclassification. In contrast, the study by Massip et al., which included mostly susceptible isolates, reported a much better performance, although they also observed an underestimation with E-test and an overestimation with VITEK 2 (14). The performances calculated on the entire collection in our study likely represent a 'worst-case scenario,' as it includes many challenging strains that are difficult to categorize. However, this type of evaluation is essential to assess the robustness of the methods under conditions where accurate classification is most critical. Notably, when focusing on the *E. coli* isolates, which are more susceptible, both VITEK 2 and disk diffusion achieved performances that met ISO standards, further demonstrating the impact of strain selection on method performance. A limitation of this study is that only one brand of MH agar was tested, and evaluating others could reveal potential differences between manufacturers.

For many antibiotics, it is common practice to determine MICs when there is doubt about the susceptibility of the pathogen. Our results confirmed that the unique relevant method to determine mecillinam MIC remains the reference agar dilution method, which is cumbersome for clinical microbiology laboratories.

This study was conducted on a collection of CPE, which inherently exhibit higher mecillinam MICs compared to a wild-type population. Indeed, among the *E. coli* isolates in this study, only one strain had a MIC of 0.5 mg/L, which corresponds to the ECOFF for this species. It indicates that CPE strains, even if susceptible, have higher mecillinam MICs than wild-type isolates. Accordingly, our results remain crucial to select the most relevant diagnostic test that will contribute to the appropriate use of pivmecillinam as a last-resort treatment for urinary tract infections caused by CPE. For use in uncomplicated urinary tract infection, as most Enterobacterales are susceptible to mecillinam, the microbiologist can safely rely on disc diffusion as demonstrated by EUCAST or Skov et al. (17).

To conclude, in a context of the rapid spread of carbapenemase-producing Enterobacterales, it is crucial to consider antimicrobial alternatives, such as pivmecillinam, which remains active on a large proportion of carbapenemase-producing Enterobacterales (8). This is all the more important, as the US Food and Drug Administration has just approved the use of pivmecillinam for the treatment of uncomplicated urinary tract infections in April 2024.

## SUPPLEMENTAL MATERIAL

Supplemental figures jcm.01473-24-s0001.docx Fig. S1 and S2.

Table S1 jcm.01473-24-s0002.xlsx Global characteristics of strains included in this study (isolation date, area of isolation, origin, and carbapenemase content) and results of inhibition zone diameters and MICs obtained.

Table S2 jcm.01473-24-s0003.xlsx Performance metrics of mecillinam susceptibility testing methods using the error-rate-bound method across different guidelines and carbapenemase types (A) and error rate acceptance limits (B).

ASM does not own the copyrights to Supplemental Material that may be linked to, or accessed through, an article. The authors have granted ASM a non-exclusive, world-wide license to publish the Supplemental Material files. Please contact the corresponding author directly for reuse.
