# Supplementary material for: Performance of disc diffusion and four commercially available MIC tests to determine mecillinam susceptibility on carbapenemase-producing Enterobacterales
Source: J Clin Microbiol. 2025 Apr 14;63(5):e01473-24. doi: 10.1128/jcm.01473-24 (PMC12077098; doi:10.1128/jcm.01473-24)
Supplement: Supplemental figures — Fig. S1 and S2. [file jcm.01473-24-s0001.docx]

**SUPPLEMENTARY FIGURES**

**Figure S1**. Distribution of mecillinam inhibition diameters (A) and MICs using agar dilution (B) for the strains included in the study. These strains were selected to have a Gaussian distribution using their inhibition zone diameters.

**Figure S2.** Distribution of MICs to mecillinam for the clinical strains included in the study according to the methods used: reference method agar dilution, Liofilchem^®^ MIC strip test, VITEK^®^ 2, and Sensititre™ broth microdilution. These distributions were compared using the Student's t-test, and asterisks indicate a significant difference with a p<0.0001.
